# The effects of irrigation on the survival of *Clostridium sporogenes* in the phyllosphere and soil environments of lettuce

**DOI:** 10.1007/s13205-024-04069-5

**Published:** 2024-09-21

**Authors:** Johannes Cornelius Jacobus Fourie, Deidre Van Wyk, Cornelius Carlos Bezuidenhout, Charlotte Mienie, Rasheed Adeleke

**Affiliations:** Unit for Environmental Sciences and Management, North-West University, Potchefstroom, South Africa

**Keywords:** *Clostridium sporogenes*, Irrigation, Phyllosphere, Soil environments, QPCR

## Abstract

This study aimed to address the gap in knowledge regarding the fate of foodborne pathogens within agro-ecosystems. It specifically focused on the surrogate microorganism *Clostridium sporogenes*, which was introduced into lettuce-producing environments via surface and spray irrigation methods, respectively. The concentration of *C. sporogenes* in the rhizosphere, phyllosphere, and non-rhizosphere soil was quantified by quantitative polymerase chain reaction (qPCR) over a 42-day trial. The surface irrigation method exhibited a more noticeable contamination effect on the soil environments, compared to the phyllosphere. The results indicated a noticeable increase in *C. sporogenes* concentrations during the initial 22 days, with a 10.4-fold rise (0.39–4.05 log copy numbers/g soil) in the rhizosphere and 1.9-fold increase (2.97–5.59 log copy numbers/g soil) in the non-rhizosphere. However, concentrations in both soil environments subsequently decreased, falling below the initial inoculum concentration by the end of the trial. In contrast, the spray irrigation method resulted in most of the contamination being localised on the lettuce phyllosphere, with a high *C. sporogenes* concentration of 9.09 log copy numbers/g leaves on day 0. This concentration exponentially decreased to a minimal 0.019 log copy numbers/g leaves by day 32. Although concentrations in both soil environments decreased over time, trace concentrations of *C. sporogenes* were detectable at the end of the trial, posing a potential hazard to the microbiological safety of postharvest produce. These findings shed light on the dynamics of *C. sporogenes* in agro-ecosystems and underscore the importance of irrigation practices that ensure the safety of those who consume fresh produce.

**Supplementary Information:**

The online version contains supplementary material available at 10.1007/s13205-024-04069-5.

## Introduction

The consumption of fresh produce has increased over the past few decades due to its accompanying health and nutritional benefits (Alam et al. 2014; Machado-Moreira et al. 2019). This has led to the need for mass distribution within the shortest amount of time to meet this growing demand and, as a result, consumers are increasingly exposed to foodborne diseases (Balali et al. 2020). Fresh, ready-to-eat (RTE) produce is likely to be contaminated with disease-causing bacteria through direct contact with soil or animal manure, or irrigation with contaminated water (Gurtler and Gibson 2022). According to Manshadi et al. (2013), wastewater or sewage-polluted surface water is used to irrigate about 10% of the world's crops. This is of concern, since most foodborne pathogens originate from the intestinal tract and faecal material of mammals (Hamza et al. 2018). The anaerobic pathogens *Clostridium botulinum* and *Clostridium perfringens* are examples of bacterial hazards; they have environmental and intestinal origins (Rai and Tripathi 2007). They impact public health and the economy through their ability to produce several deadly toxins and spores that are highly resistant to environmental stressors, which contributes to their survival, pathogenicity, and transmission (Alegbeleye et al. 2018; Hoffman et al. 2015; Li et al. 2016; Palmer et al. 2019).

Because approximately 70% of the world's fresh water is used for irrigation, the quality of these water sources is of great importance (Scanlon et al. 2007). In South Africa, routine testing for *Clostridium* pathogens in water sources used for irrigation is not an established practice. A previous study conducted by the authors reported high levels of *Clostridium* pathogens in several surface water systems used for irrigation in the North West Province (Fourie 2017). When the genomic features of the pathogenic *C. perfringens* found in these systems were investigated, they exhibited multidrug resistance, enhanced virulence factors, and environmental adaptation through mobile genetic elements (Fourie et al. 2020). Based on these hazards, the present study focusses on the potential spread of *Clostridium* in the food chain. It is hypothesised that when plants are irrigated with contaminated water, *Clostridium* species can establish and survive in RTE vegetables, such as lettuce, and are able to become endophytic. However, field studies that involve pathogenic *Clostridium* are very restricted due to environmental health and safety concerns (Park et al. 2018). Therefore, to assess how irrigation water transmits *Clostridium* in the preharvest environment of lettuce, a surrogate species, namely *Clostridium sporogenes*, was used in a greenhouse setting. To investigate its survival in the rhizosphere, phyllosphere, and non-rhizosphere soil of lettuce, the concentration of *C. sporogenes* was determined by using a qPCR assay.

## Materials and methods

### Experimental layout

A design plan (Figure S1) for the greenhouse trial was followed, which comprised three different treatments. Lettuce seedlings were transplanted into pots containing sterilised soil. Treatment 1 and 2 inoculated *Clostridium sporogenes* into the lettuce-producing environment through simulating surface and spray irrigation, respectively. Treatment 3 served as a control treatment, with no *Clostridium sporogenes* present in irrigation water. Twenty-five replicate pots were used for each treatment. Pots were grouped by treatments to avoid cross-contamination. The trial was conducted at Eco-Rehab in Potchefstroom, South Africa. The biosafety level 1 (BSL-1) greenhouse is equipped with temperature control, LED grow lights, and HEPA filters (Figure S2). The greenhouse maintained an average temperature of 26 °C, with a 16:8 h light–dark cycle for the duration of the experiment.

### Bacterial strain, growth conditions, and inoculum preparation

For this study, the *Clostridium sporogenes* ATCC 3584 strain was purchased from Microbiologics (Minnesota, USA). The inoculum was firstly grown anaerobically on cooked meat medium (Oxoid, UK) at 37 °C for 24 h to confirm culture purity. Spores of *Clostridium sporogenes* were used as inoculum and harvested through a modified method described by Rabi et al. (2017). In short, a single colony was used to inoculate brain heart infusion (BHI) broth (Oxoid, UK) and incubated using a rotary shaker at 100 rpm at 35 °C under anaerobic atmosphere conditions for 48 h. Endospore staining was then performed to determine spore maturation. The spores that were released from the mother cells were collected by centrifugation (4000 rpm, 15 min); after that, they were washed three times with 50 mM phosphate-buffered saline (PBS, pH 7.3). After each wash, vegetative cells and other cellular debris were removed from the top of the spore pellet to obtain a pure spore suspension. The concentration of spores was estimated by spore staining, followed by direct microscopic count (DMC) (~ 3 × 10^11^ CFU/ml) and then stored at 4 °C until they were used for inoculation.

### Soil collection, lettuce preparation, and sterilisation

Agricultural soil was collected from the Agricultural Research Council: Grain and Crop Institute in Potchefstroom, South Africa. The soil composition was determined by the particle size distribution of soil, as described by Gerber et al. (2015); the results showed that the soil consisted of 39% sand, 30.4% silt, and 29% clay. Based on its composition, the soil was classified as loamy clay soil (Figure S3). The soil was then sterilised through autoclaving it twice (121 °C, 30 min). Seedlings of ‘Great Lakes’ lettuce (*Lactuca sativa* ‘Great Lakes’) were purchased from a local nursery in Potchefstroom, South Africa. The soil surrounding the roots of the seedlings was removed, washed with sodium hypochlorite (NaOCl 10%; v/v), and rinsed twice with distilled H_2_O. The sterilised soil as well as the leaves and roots of the lettuce was then assessed by conventional PCR methods to ensure the absence of *C. sporogenes.*

### Irrigation of soil and lettuce with *C. sporogenes*

Each lettuce seedling was transplanted into a separate pot (23 cm diameter) that contained 2 kg of sterilised soil. Three different treatments followed, each in separate areas to prevent any cross-contamination. Treatment 1 simulated surface irrigation: the soil was carefully covered with 100 ml of distilled water containing *C. sporogenes*, limiting any contact between the water and the leaves of the lettuce seedling. Treatment 2 simulated spray irrigation: the leaves were sprayed with a hand-held spray bottle containing 100 ml of distilled water and *C. sporogenes.* Treatment 3 served as the negative control group: 100 ml of distilled water was used to irrigate the soil containing the seedling. The bacterial suspension in irrigation water used for treatments 1 and 2 was measured by using spore staining, followed by DMC and was approximately 9 × 10^9^ CFU/ml for both treatments. Each pot used throughout the trail was placed onto a plastic tray and provided bottom watering with distilled water to ensure the soil and lettuce plants were not disturbed during the trial. Distilled water was collected from the laboratory; the pots were watered daily for the first 7 days; thereafter, they were watered every 3 days or when the soil surfaces began to dry. A half-strength Hoagland’s solution was also added weekly via bottom watering (Hoagland and Arnon 1950).

### Sample collection

Rhizosphere, non-rhizosphere soil, and phyllosphere samples were aseptically collected from each treatment on days 0, 9, 22, 31, and 42. To collect phyllosphere samples, the edible leaves of lettuce were removed by cutting off the whole head of the lettuce with a sterile blade and placing them in sterile plastic bags. Rhizosphere soil was collected by removing the lettuce plant from the soil, gently shaking it, and collecting the soil from the roots. Non-rhizosphere soil was obtained with a sterile spoon. The samples were collected at 7 cm away from the lettuce and 5 cm below the soil surface. All soil samples were collected in separate 50 ml sterile falcon tubes. All samples were immediately transported to the laboratory for further processing and analysis.

### Sample preparation, DNA extraction, and qPCR

For the phyllosphere samples, the outermost leaves of each lettuce head were removed and discarded. Five leaves were then chosen at random from the remaining lettuce head layers. These were aseptically ground` with a pestle and mortar to ensure more uniform leaf samples across all treatments. Total DNA was then extracted from 1 g of the leaf homogenate by using the DNeasy Plant Pro Kit (Qiagen, DE), following the manufacturer’s instructions. For the soil samples, each collected sample was aseptically sieved (3 mm) to ensure thorough homogenisation before DNA extraction. The total DNA was then extracted from 1 g of soil using the NucleoSpin^®^ Soil kit (Macherey–Nagel, GE), also following the manufacturer’s instructions. A NanoDrop spectrophotometer and a 1% agarose gel electrophoresis were used to evaluate the concentrations and integrity of the DNA extracted from both leaf and soil samples (Thermo Scientific, USA). All DNA samples were stored at − 20 °C for downstream analysis.

Absolute quantifications were done by qPCR assay, using the QuantStudio 5 Real-Time PCR System (Applied Biosystems, USA) with SYBR^®^ Green fluorescence reagent. Previously described *C. sporogenes* primers were chosen for the detection and quantification of a 96 bp fragment of the *ger*AA gene (Morandi et al. 2015). The primer sequences are as follows: *ger*AA-F CCG CAG GAA TAA ACA ATG TTC TAA and *ger*AA-R CAG CAT AAG CAG CCC CTA AAA. All reactions were performed in a final volume of 25 µl and consisted of 10 µl of 2 × PowerUp™ SYBR Green Master Mix (Applied Biosystems, USA), 1 µl of each primer (0.4 µM each), 11 ul of nuclease-free water and 2 µl of gDNA. The Green Master Mix comprised SYBR Green I dye Dual-Lock Taq DNA Polymerase, dNTPs mix with dUTP/dTTP, heat-labile UDG, passive reference dye ROX, and buffer components. The reactions were carried out in 96-well plates sealed with film. The thermal cycling conditions that were used are as follows: initial denaturation at 95 °C for 3 min, followed by 40 cycles at 95 °C for 15 s, and 60 °C for 60 s. All runs included a non-template negative control and *Clostridium sporogenes* ATCC 3584 as the positive control.

### Standard curve construction

To construct the standard curve, genomic DNA from a pure culture of *C. sporogenes* (37 ℃, 24 h) was extracted using the Quick-DNA Fungal/Bacterial Miniprep Kit (Zymo Research, USA). This was followed by qPCR amplification of tenfold serial dilutions of the pure gDNA, with concentrations ranging from 10 ng to 1 × 10^–5^ ng of gDNA. The data was initially analysed with QuantStudio™ Design and Analysis Software (Version 1.3.1) at a threshold determination of 0.03. Threshold cycle (Ct) values were plotted against the corresponding concentration of each DNA dilution to assess the linear range of detection and reliability of the qPCR assay. The standard curve was calculated as *y* =  − *ax* + *b* (a refers to the standard curve slope and b refers to the y-intercept). The amplification efficiency of the reaction (*E*) was calculated as E = (10^−1/a^), and the percent efficiency was evaluated as (*E* − 1) × 100%. According to Pfaffl and Bustin (2004) and Rutledge and Côté (2003), amplification efficiencies of standard curves between 90 and 110%, which correspond to a slope between − 3.1 and − 3.6, and coefficient of determination (*R*^2^) > 0.98 are considered to be reliable comparison trend lines for the quantification of unknown samples. For all the analyses, three technical replications of each sample were performed.

### Data analysis

All qPCR data was initially analysed with QuantStudio™ Design and Analysis Software (Version 1.3.1), where the Ct values were exported into Microsoft Excel Worksheet for further statistical analysis. An analysis of variance (ANOVA) was conducted to determine statistically significant differences within and between the two irrigation methods (surface and spray irrigation) on *C. sporogenes* copy numbers/g of soil or leaves. A p value of 0.05 for the ANOVA analysis was considered as significant.

## Results

### Sensitivity, standard curve, and amplification efficiency of qPCR assay

To detect and quantify *C. sporogenes* in environmental samples, a qPCR assay was utilised. The performance and sensitivity of the assay (Figure S4) were evaluated by amplifying the *ger*AA gene in a tenfold serial dilution of pure gDNA at known concentrations (10 ng – 1 × 10^–5^ ng). The amplification curve showed good reproducibility, and the fluorescence intensity changed consistently with the serial dilution of the gDNA concentration. The results of the constructed standard curve showed a strong linear relationship between the Ct values and the known DNA concentrations over seven orders of magnitude. The coefficient of determination (*R*^2^) was high at 0.996, indicating a good fit of the data points on the standard curve. The slope of the log-linear phase was − 3.51. The Y-intercept was 25.706, and the amplification efficiency (*E*) was estimated to be 93.1%. According to the melting curve, the 96 bp amplicons that were generated were unimodal and showed a single dissociation peak at 72.1 ± 0.09 °C, indicating that the primers used in this study exhibited adequate specificity. Additionally, no melting curve was detected in the negative controls.

### Survival of *C. sporogenes* in soil, rhizosphere, and phyllosphere of lettuce

**Fig. 1:**
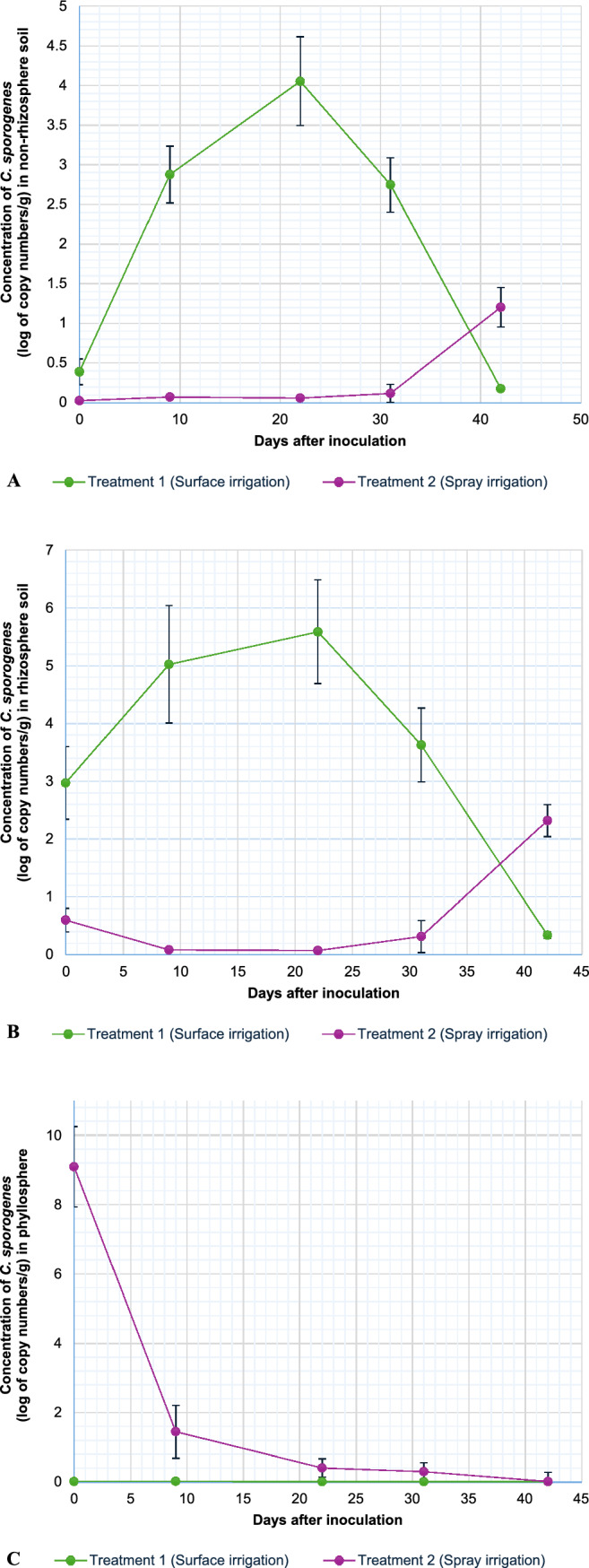
Survival of *C. sporogenes* ATCC 3584 in non-rhizosphere soil (**A**), rhizosphere (**B**), and phyllosphere (**C**) of lettuce over 42 days following two different irrigation treatments. Treatment 1 introduced *C. sporogenes* into the lettuce-producing environments via surface irrigation (green); whereas Treatment 2 did so by spray irrigation (purple). Both treatment methods administered a single 100 ml dose of *C. sporogenes* (9 × 10^9^ CFU/ml) on day 0. Afterward*, Clostridium sporogenes* concentrations were determined in each of the three lettuce environments on different days (0, 9, 22, 31, and 42 days) by means of qPCR; these are reported as log copy numbers/g sample. The results are the means and standard deviation of five replicates

Based on the constructed standard curve (Figure S4), the qPCR assay was able to detect and quantify *C. sporogenes* in the non-rhizosphere soil, rhizosphere, and phyllosphere of lettuce after being contaminated with surface (T1) and spray (T2) irrigation over the 42-day duration of this study. *Clostridium sporogenes* was immediately detectable in leaves and soil samples at various concentrations after the inoculated water was applied. The initial concentrations of 2.97 and 0.6 log copy numbers/g soil were reported in non-rhizosphere soil (Fig. 1A), 0.39 and 0.03 log copy numbers/g soil in rhizosphere soil (Fig. 1B), and 0.02 and 9.09 log copy numbers/g leaves (Fig. 1C) after surface and spray irrigation, respectively. However, the DNA extracted from the soil and leaf samples from the control treatment (T3) showed no amplification of the *ger*AA gene at any of the sampling days, which was expected.

The detectable amount of *C. sporogenes* in the non-rhizosphere over the 42 days using qPCR (Fig. 1A) was significantly different between the two irrigation treatments (Table 1). For treatment 1, the concentration of *C. sporogenes* showed an increase, reaching a peak of 5.59 log copy numbers/g soil on day 22. However, the concentration gradually decreased to below the initial concentration (2.98 log copy numbers/g soil) at day 42 (0.34 log copy numbers/g soil). The opposite trend was observed for treatment 2, where, over the first 9 days, the concentration decreased from 0.6 to 0.08 log copy numbers/g soil. Following this, the *C. sporogenes* concentrations remained relatively consistent, only showing a 7.25-fold increase from day 31 (0.32 log copy numbers/g soil) to day 42 (2.32 log copy numbers/g soil).

**Table 1:** Significant differences between and within treatments 1 and 2 for *Clostridium sporogenes* concentration (log copy numbers /g) in the soil environments and phyllosphere of lettuce over a 42-day trial period

One-way ANOVA	Day				
Between treatments	0	9	22	31	42
T1-P vs T2-P	< 0.001*	0.005*	0.019*	0.009*	0.976
T1-R vs T2-R	< 0.001*	< 0.001*	< 0.001*	< 0.001*	< 0.001*
T1-S vs T2-S	< 0.001*	< 0.001*	< 0.001*	< 0.001*	< 0.001*
Within treatment 1					
T1-P vs T1-S	< 0.001*	< 0.001*	< 0.001*	< 0.001*	< 0.001*
T1-P vs T1-R	< 0.001*	< 0.001*	< 0.001*	< 0.001*	< 0.001*
T1-R vs T1-S	< 0.001*	< 0.001*	0.025*	0.021*	< 0.001*
Within treatment 2					
T2-P vs T2-S	< 0.001*	0.006*	0.041*	0.970	< 0.001*
T2-P vs T2-R	< 0.001*	0.005*	0.035*	0.109	< 0.001*
T2-R vs T2-S	< 0.001*	0.066	0.009*	0.184	< 0.001*
Two-way ANOVA					
T1–T2-P vs T1–T2-S	< 0.001*	< 0.001*	< 0.001*	< 0.001*	< 0.001*
T1–T2-P vs T1–T2-R	< 0.001*	< 0.001*	< 0.001*	< 0.001*	< 0.001*
T1–T2-R vs T1–T2-S	< 0.001*	< 0.001*	0.015*	0.074	< 0.001*
T1-P-S-R vs T2-P-S-R	< 0.001*	< 0.001*	< 0.001*	< 0.001*	< 0.001*

*T1* treatment 1 (surface irrigation), *T2* treatment 2 (spray irrigation), *P* phyllosphere of lettuce, *S* non-rhizosphere soil, *R* rhizosphere soil

^*^Means they are significantly different from each other (p < 0.05)

When compared, the survival of *C. sporogenes* in the rhizosphere soil (Fig. 1B) followed a similar pattern to that of the above-mentioned non-rhizosphere soil (Fig. 1A). Different trends were observed in the rhizosphere soil of the two irrigation methods. The concentration of *C. sporogenes* in the rhizosphere soil of lettuce following treatment 1 showed an initial 10.4-fold increase from day 0 to 22 (0.39–4.05 log copy numbers/g soil) and then a decrease in concentration at day 31 and 42 (2.75 and 0.18 log copy numbers/g soil, respectively). The prevalence of *C. sporogenes* following treatment 2 remained consistently low in the rhizosphere soil for the first 3 weeks of the study, with concentrations of 0.03–0.06 log copy numbers/g soil for days 0–22, respectively. However, the concentration increased tenfold during the last 2 weeks of the study, reaching a high of 1.2 log copy numbers/g soil at day 42.

The initial concentration of *C. sporogenes* in the phyllosphere samples (Fig. 1C) varied greatly between the two irrigation methods: the spray irrigation method used in treatment 2 resulted in a much higher detectable amount on the leaves (9.09 log copy numbers/g leaves) than that of the surface irrigation used in treatment 1 (0.018 log copy numbers/g leaves). However, in treatment 2, the concentrations drastically changed from 9.09 to 1.45 log copy numbers/g leaves during the first 9 days, resulting in a 6.3-fold decrease. The concentration of *C. sporogenes* then continued to gradually decrease until day 42, where only 0.019 log copy numbers/g leaves remained. Additionally, low concentrations of *C. sporogenes* were also detected on the phyllosphere of lettuce following surface irrigation, ranging between 0.012 and 0.02 log copy numbers/g leaves throughout the 42-day trial.

Table 1 shows the statistically significant differences between the treatments and lettuce environments. Treatment 1 was significantly different from treatment 2 in both soil environments over the 42-day trial. However, when comparing treatments 1 and 2, the phyllosphere showed initial significant differences up to day 31, whereas there was no significant difference between the two treatments, as both concentrations reduced to 0.01 log copy numbers/g leaves at day 42. Furthermore, statistical differences were observed over the entire duration of the trial among all three lettuce environments from treatment 1. Similar observations were made within treatment 2, except for day 31, with no statistical differences among the concentrations in the non-rhizosphere soil, rhizosphere, and phyllosphere. A two-way ANOVA found that the concentrations in all three environments for both treatments were statistically significant throughout the trial. This means that the method of irrigation (surface or spray irrigation) influenced the transport and survival of *C. sporogenes* in the non-rhizosphere soil, rhizosphere soil, and phyllosphere of lettuce.

## Discussion

Many of the studies that investigated the microbial contamination of vegetable crops adopted a spiking approach to illustrate the potential hazard associated with the consumption of raw vegetables. In such instances, harvested vegetables are spiked with a known concentration of microbial contaminants. This approach does not yield evidence of the potential endophytic attributes of such contaminants, which may confirm their abilities to colonise and form symbiotic partnerships with the host plant. Therefore, for this study, the researchers irrigated the lettuce plants with contaminated water; this method allowed the researchers to monitor the development of the plant–microbe interaction over a period of time and establish the colonisation of the lettuce by the surrogate indicator organism *Clostridium sporogenes*. Furthermore, the researchers were able to investigate the fate of the *Clostridium* in the rhizosphere, phyllosphere, and non-rhizosphere soil, following surface and spray irrigation in a greenhouse setting. A culture-independent approach, using qPCR, was used to determine the survival of *C. sporogenes* in these three environments. Furthermore, the qPCR technique enabled the detection and quantification of the *ger*AA gene present in *C. sporogenes* in all three of the investigation areas. The use of qPCR has been shown to successfully quantify *Clostridium* species in processed vegetables, milk, and dairy products (Chon et al. 2012; Morandi et al. 2015; Sahiner et al. 2022), with great sensitivity and accuracy (MacDougall et al. 2018). The current study successfully achieved a comprehensive quantification of *C. sporogenes* by exclusively using DNA extracted from agriculturally intricate niches, which are recognised for their complexity and susceptibility to diverse biotic and abiotic influences (Wydro 2022). This methodology facilitated an in-depth exploration into the fate of *Clostridium* within these three environments subsequent to a simulated contamination event induced by irrigation.

Different irrigation methods can greatly affect the degree of microbial contamination on receiving produce. Several studies report that the probability of contaminating the foliage of produce is increased by using overhead sprinkler irrigation (Ganeshan 2015; Iwu and Okoh 2019; Kisluk and Yaron 2012). These studies also suggest that the use of irrigation methods that limit the contact between the water and the produce phyllosphere may decrease the risk of contamination, examples include surface irrigation. A possible explanation for the *C. sporogenes* on the phyllosphere samples that received surface irrigation in this study could be the backsplash of contaminated water or contact with soil during the initial irrigation treatment (Ibekwe et al. 2009). Alternatively, endophytic colonisation of fodder plants with other *Clostridium* species, such as *C. botulinum,* has been reported. However, further research is needed regarding this occurrence. *Clostridium* proliferates in environments such as soil, but little is known about its role as endophytic or plant-associated bacteria (Zeiller et al. 2015). The results of the current study also show an initial high level of *C. sporogenes* detected on the phyllosphere of lettuce after spray irrigation. However, these levels show a drastic decrease over time. A similar study by Ercolani (1997) also report a decline in levels of *C. pasteurianum*, *C. perfringens,* and *C. sporogenes* in the leaves of tomato and basil plants after spray inoculation. This is because spray irrigation covers the crop and the soil with water. The majority of the water that contained *C. sporogenes* was retained on the phyllosphere, resulting in a lesser amount of bacteria being introduced directly into the soil through droplets.

It is important to state that the aerial portion of a plant is a harsh and unstable environment for most microorganisms (Chaudhry et al. 2021), especially for obligate anaerobes, such as *Clostridium* species. It is, therefore, expected that *C. sporogenes* on leaves would have a higher death than growth rate. The survival and, subsequently, the colonisation of microorganisms on produce are influenced by direct exposure to multiple environmental factors, such as oxygen, temperature, water availability, and UV radiation, as is the case with endophytes (Alegbeleye et al. 2022; Chaudhry et al. 2021). The nutrient sources on leaves are also sparse in comparison to other environments, such as the rhizosphere soil or the enteric environment of mammals (Chaudhry et al. 2021; Leveau and Lindow 2001). Although the spores produced by *C. sporogenes* are tolerant to many of these environmental stressors, studies have shown that the presence of oxygen and the lack of essential nutrients, such as L-alanine, greatly affects the germination success of *Clostridium* spores (Fujioka and Frank 1966; Wang et al. 2017). Although *C. sporogenes* was unable to colonise the phyllosphere of lettuce successfully through the two irrigation methods in this study, it was still detected in low levels after 42 days. If *Clostridium* species and their spores are present on produce during preharvest, it could jeopardise the postharvest quality and pose a safety hazard for consumers. This is evident in studies that evaluated the microbiological safety of minimally processed RTE vegetables and salads, including spinach, mixed leaf salad, and lettuce that have shown the presence of pathogenic *Clostridium* (Bakri et al. 2009; Eckert et al. 2013).

Regarding the subterranean survival of *C. sporogenes*, it is important to note that even though *Clostridium* species are ubiquitously distributed in soil, it does not imply that they can survive in any soil. Soil structures and soil types greatly affect the prevalence, survival, and movement of bacteria in soil (Palmer et al. 2019). Fine-grained soil, such as the clay loam soil used in this study, has high nutrient values and water-retaining properties that could aid the survival of some enteric pathogens (Jamieson et al. 2002). This is likely the case with *Clostridium* species as well. When pathogens are introduced via water to the topsoil horizon, the water translocates downward through the pore structures of the soil; this disperses bacteria to lower soil horizons, such as the rhizosphere and non-rhizosphere soil (Gessler and Bohnel 2006). In the current study, after the water translocated, the concentrations of *C. sporogenes* in rhizosphere and non-rhizosphere soil showed similar trends in both irrigation treatments. This indicates that the rhizosphere soil environment does not provide better or worse conditions for the proliferation of *C. sporogenes* in comparison with non-rhizosphere soil. Based on the physiology of *Clostridium* species, particularly their nutrient and anaerobic requirements, the soil environments are comparatively less hostile than that of the phyllosphere (Palmer et al. 2019). There is greater availability of organic substrates and lower oxygen levels in the rhizosphere soil than in the phyllosphere, which could account for the survival of *C. sporogenes* in the rhizosphere and surrounding soil. Plant roots modulate anaerobic respiration in soil by consuming the oxygen that is present, thereby, increasing the anaerobic volume in soil and establishing oxygen-free zones (Lecomte et al. 2018). Roots also release more than 25% of their organic matter and exude nutrients, such as carbon sources, at the tips and junction of lateral roots, creating energy-rich micro-pockets/environments where anaerobic bacteria can grow and multiply (Brandl et al. 2004; Jaeger et al. 1999). The aforementioned may be a result of the delayed germination of *C. sporogenes* spores during treatment 2 of this study, which resulted in an increase in the concentration after day 31 of the trial. The *Clostridium* spores initiate germination when they sense the presence of germinants. These germinants are small molecules and may be nutrient signals from the plant roots or the surrounding soil. This enables a signalling cascade that causes the spore membrane and cortex to become degraded, which results in germination (Shen et al. 2019).

When *C. sporogenes* was introduced into the soil through surface irrigation, it resulted in higher concentrations than that of spray irrigation. Surface irrigation causes the water to cover the soil surface completely; the water then siphons through the soil matrix to reach the roots of the plant (Pachepsky et al. 2011). In this study, *C. sporogenes* was detectable in both rhizosphere and non-rhizosphere soil after surface irrigation with contaminated water. Although the concentration of *C. sporogenes* showed an initial increase over the first 22 days in both soil environments, the concentration decreased to below the original inoculation concentration at day 42. The observed trend may be attributed to the use of sterilised agricultural soil. A study done by Garcia and McKay (1969) found a similar growth and survival pattern of *C. septicum* in sterilised soil over 32 days. According to Li et al. (2019), the use of sterilised soil results in the recolonisation of a healthier soil microbiome after 6 weeks. Their results suggest that the destruction of the native microbial population brought about by soil sterilisation can rapidly recover due to the microbial activities associated with a plant. In this study, the lack of microbial competition could explain the initial increase of *C. sporogenes* in the soil. However, *C. sporogenes* could not compete with the development of a new, healthier microbiome seeded by the lettuce endophytes over the weeks and started to decrease in concentration.

## Conclusion

The use of a surrogate microorganism, namely *C. sporogenes*, showed that irrigation water is an important transmission route for *Clostridium,* which enables pathogens to proliferate in neighbouring soil environments. *Clostridium sporogenes* was detectable in the rhizosphere, phyllosphere, and non-rhizosphere soil at every sampling interval over the 42 days, demonstrating the likelihood and persistence of bacterial contamination from irrigation water in these three environments over time. However, the introduction of *C. sporogenes* into the agricultural environment through surface and spray irrigation yielded significantly different results. The application of surface irrigation resulted in much higher *C. sporogenes* contamination in soil environments; whereas, contamination from spray irrigation was predominately on the phyllosphere of lettuce. Although *C. sporogenes* was not able to successfully colonise the various soil and plant environments, rhizosphere and non-rhizosphere soil did provide a more favourable environment for it to survive and proliferate. Because this study was conducted in a controlled greenhouse setting, the simulated conditions are not a complete representation of the complex agro-ecosystem found in open fields. The fate of *Clostridium* can be influenced by external factors, such as weather, UV exposure, and temperature, as well as competition from other microorganisms that are present in these environments. However, the fact that *C. sporogenes* persisted in these three environments highlights the impact that agricultural practices, such as irrigation methods, can have on the spread of foodborne pathogens during preharvest cultivation. Additionally, qPCR has proven to be a useful method for monitoring *C. sporogenes* in various agricultural sources and should be considered for similar future studies. More attention should be given to pathogenic spore-forming bacteria, such as *Clostridium* in agro-ecosystems.

## Supplementary Information

Below is the link to the electronic supplementary material.

Supplementary file1 (DOCX 1158 KB)

## Data Availability

The original data are available upon request to the corresponding author.
