# Supplementary material for: The effects of irrigation on the survival of Clostridium sporogenes in the phyllosphere and soil environments of lettuce
Source: 3 Biotech. 2024 Sep 21;14(10):239. doi: 10.1007/s13205-024-04069-5 (PMC11415320; doi:10.1007/s13205-024-04069-5)
Supplement: Supplementary file 1 — Supplementary file1 (DOCX 1158 KB) [file 13205_2024_4069_MOESM1_ESM.docx]

**Supplementary Information (SI)**

**Title:** The establishment and survival of *Clostridium sporogenes* in the phyllosphere and soil environments of lettuce

**Proposed new title:** The effects of irrigation on the survival of *Clostridium* *sporogenes* in the phyllosphere and soil environments of lettuce

**Journal:** 3 Biotech


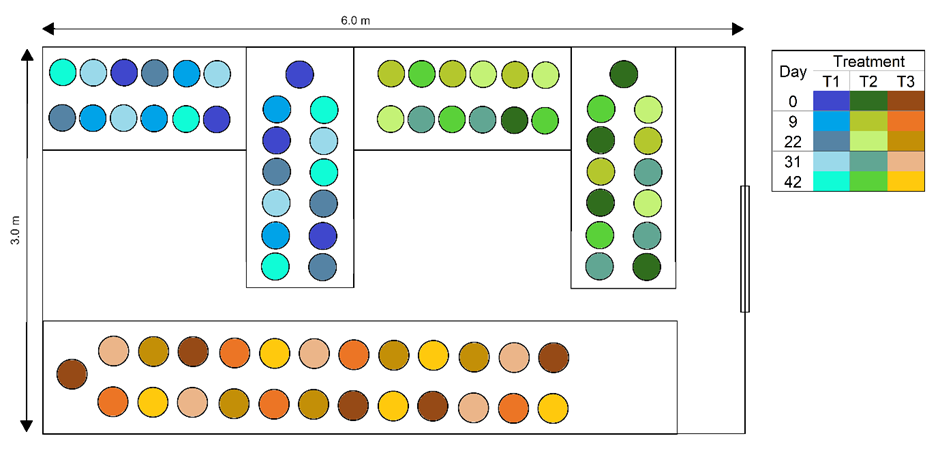


Figure S1: Layout of experimental design with different treatments (T1, T2 and T3) at Eco-Rehab greenhouse, where T1: surface irrigation, T2: spray irrigation, T3: control. Trail ran for 42 days, where sampling of rhizosphere, non-rhizosphere soil, and phyllosphere of lettuce was done on days 0, 9, 22, 31 and 42. Each sampling run consisted of 5 replicates for each lettuce environment.


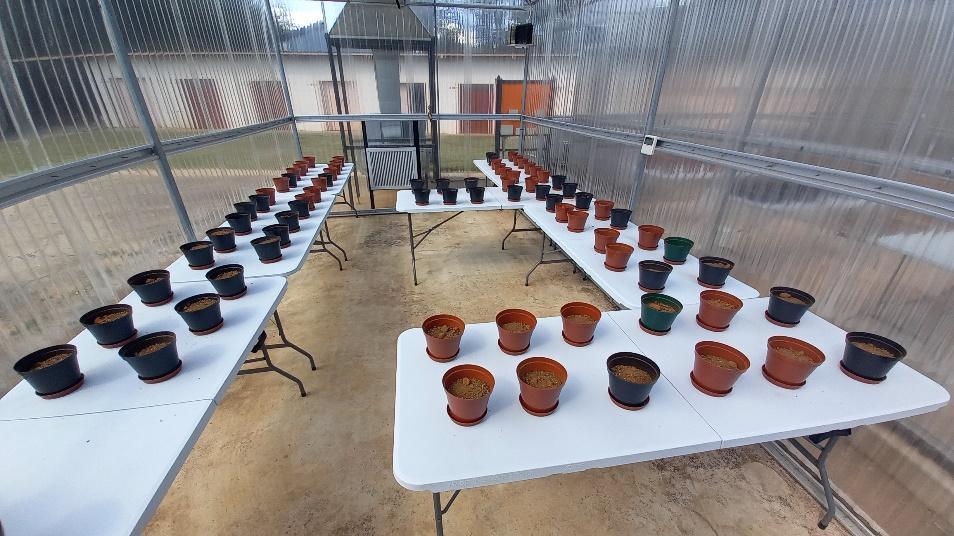

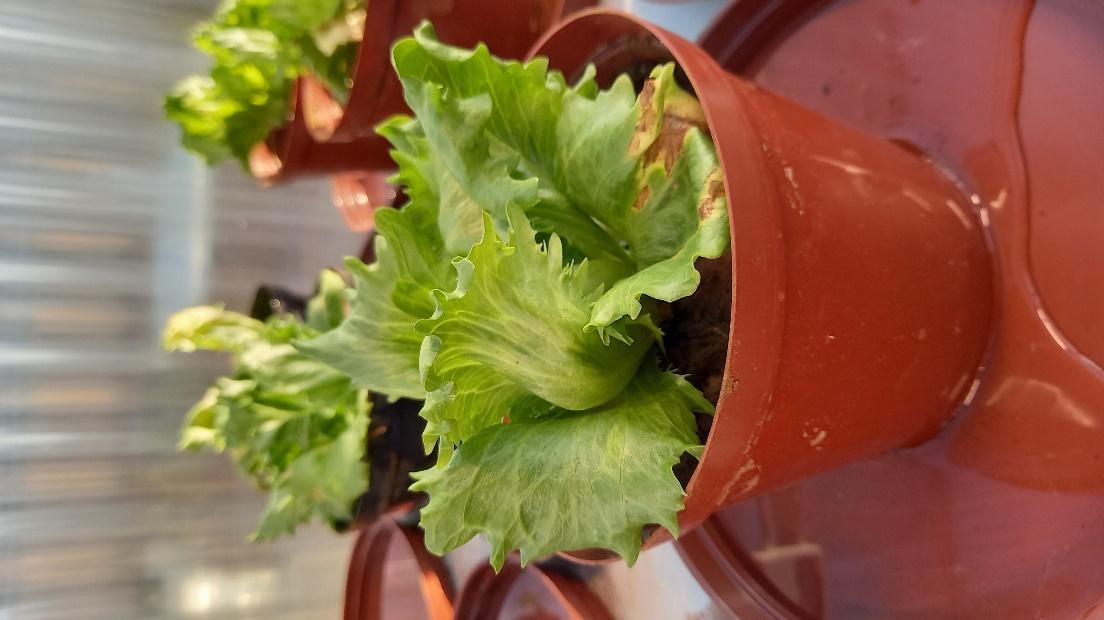

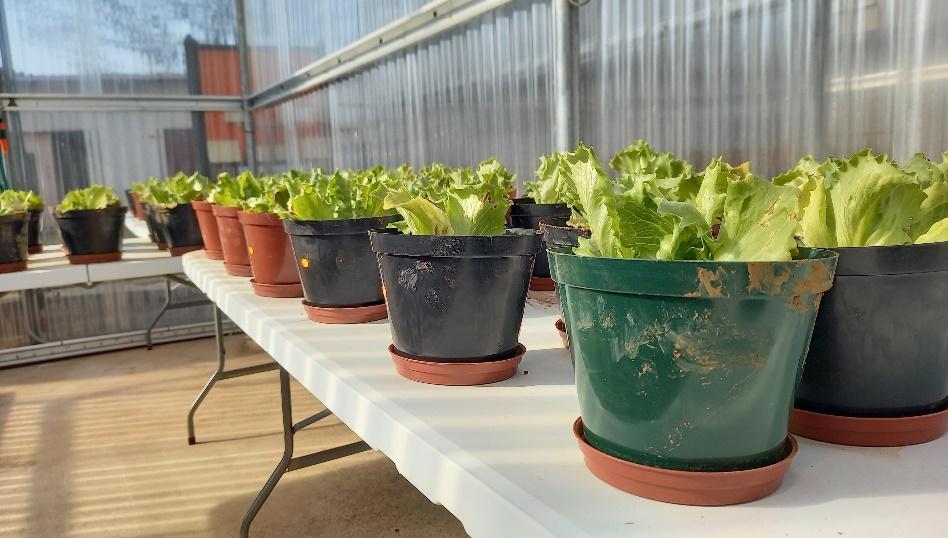

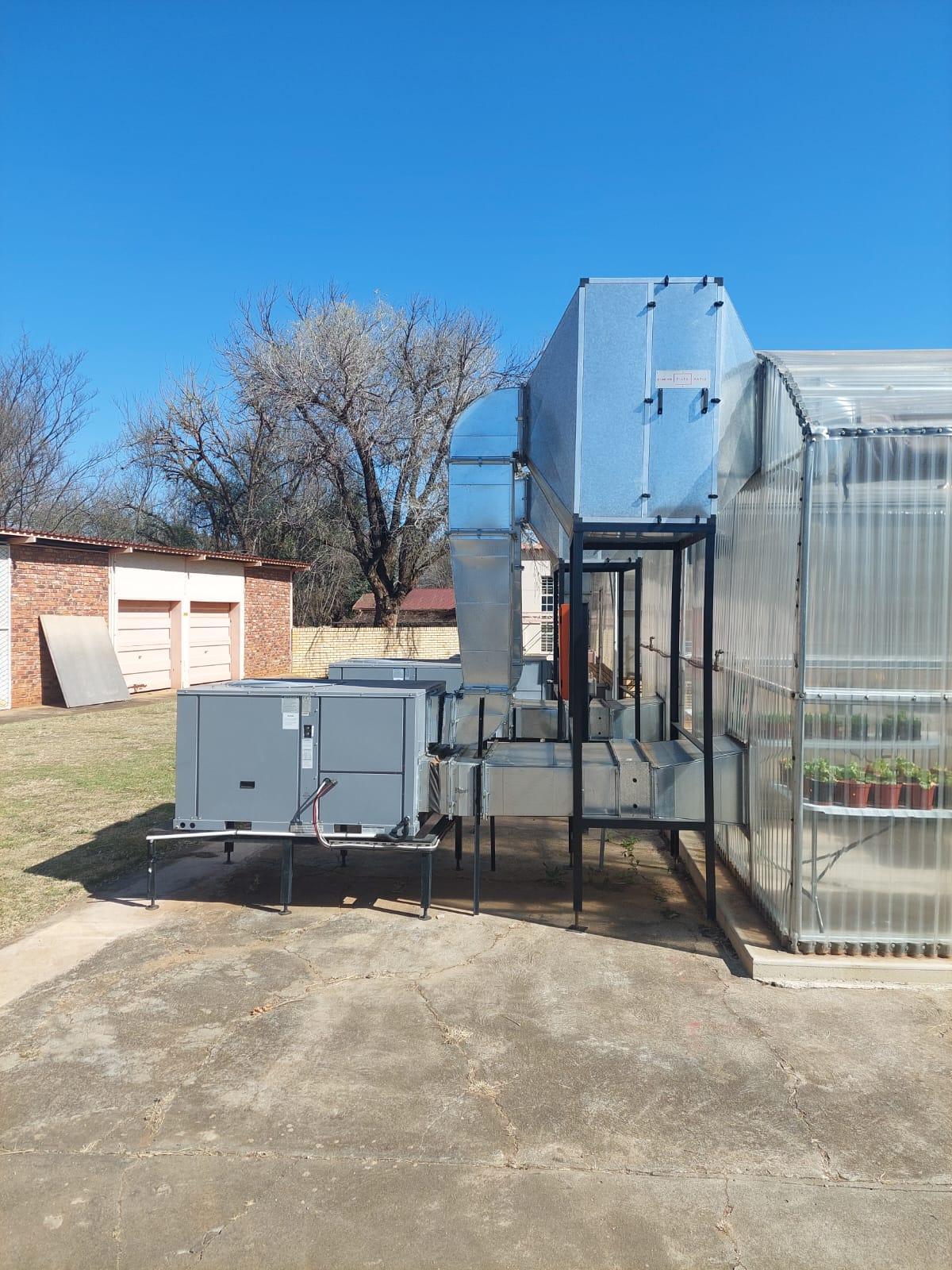



Table S1: Particle size measurments of soil used in this study via sieves with different mesh diameters.

| **Soil sample** | **Mass before shaking (g)** | **Sand** | | **Silt** | | **Clay** | | **Total mass after shaking (g)** | **Loss (g)** | **% Sand** | **% Silt** | **% Clay** |
| --- | --- | --- | --- | --- | --- | --- | --- | --- | --- | --- | --- | --- |
|  |  | **Sieve 2,0 M (g)** | **Sieve 853' M (g)** | **Sieve 707' M (g)** | **Sieve 250 µm (g)** | **Sieve 50 µm (g)** | **Bottom (g)** |  |  |  |  |  |
| Rep 1 | 100,32 | 13,04 | 20,57 | 3,80 | 28,74 | 29,58 | 2,42 | 98,15 | 2,17 | 33,61 | 32,54 | 32,00 |
| Rep 2 | 100,09 | 25,66 | 14,99 | 3,18 | 27,18 | 26,19 | 1,49 | 98,69 | 1,40 | 40,65 | 30,36 | 27,68 |
| Rep 3 | 100,07 | 24,97 | 18,05 | 3,45 | 24,81 | 25,01 | 2,22 | 98,51 | 1,56 | 43,02 | 28,26 | 27,23 |
| **Mean** | **100,16** | **21,22** | **17,87** | **3,48** | **26,91** | **26,93** | **2,04** | **98,45** | **1,71** | **39,09** | **30,39** | **28,97** |


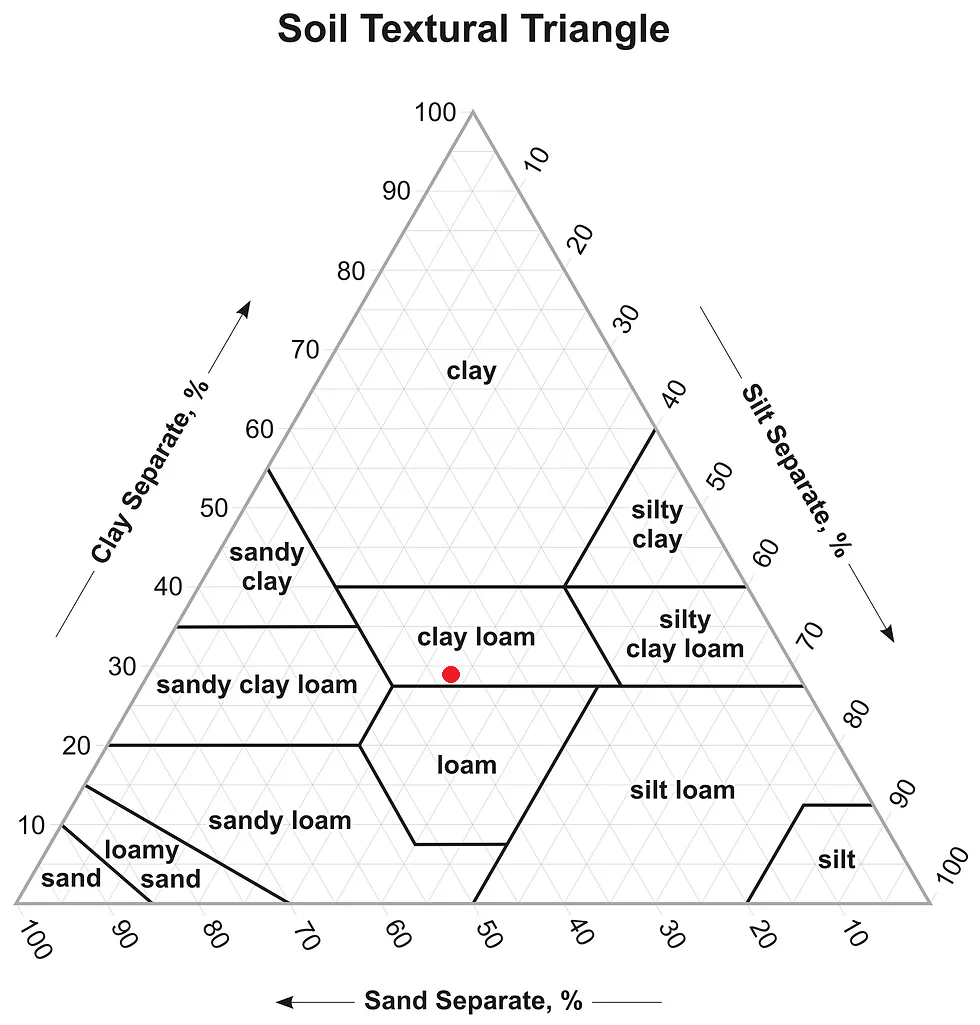


**A
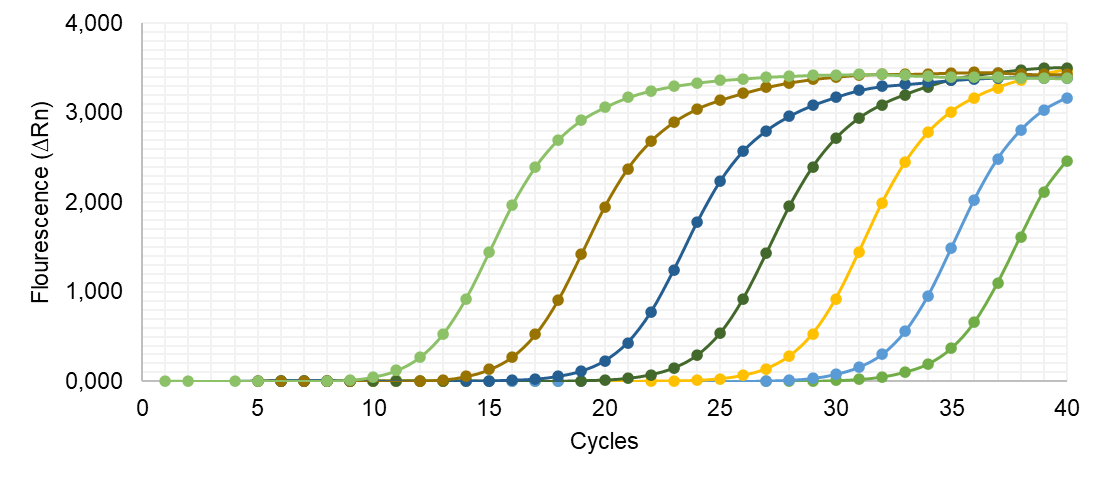
**



**B
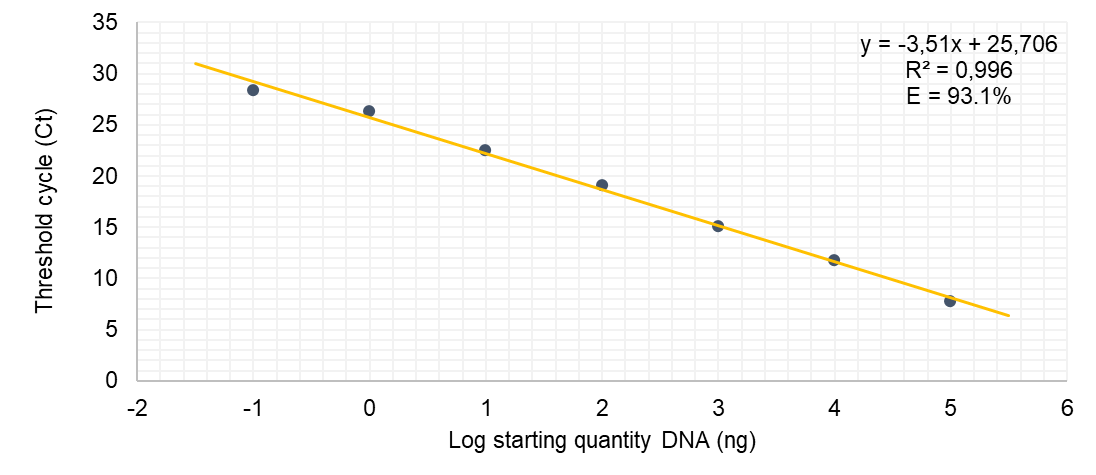
**

**C
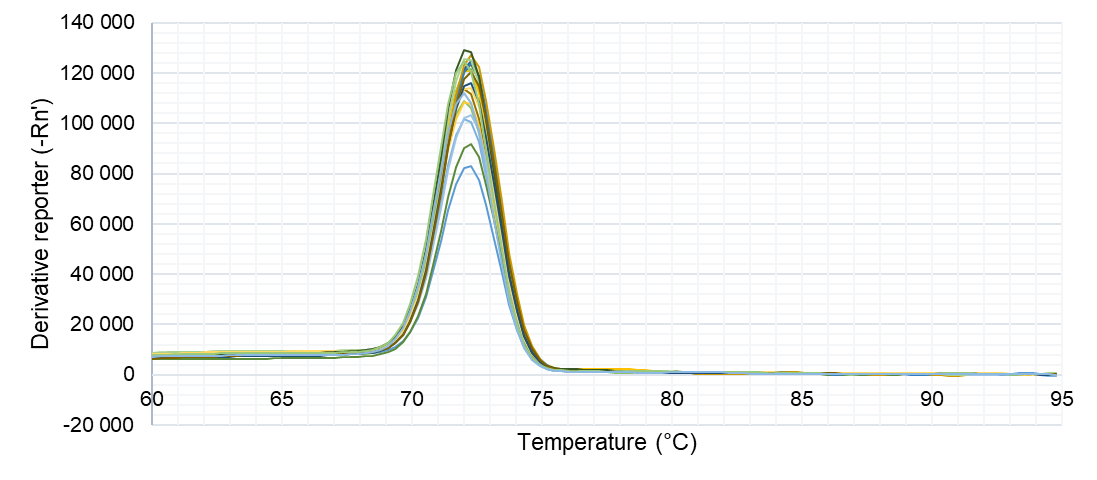
**
